# Unveiling the Mechanism of Principal Drugs of Lianpu Drink on Chronic Gastritis by Network Pharmacology

**DOI:** 10.1155/2021/5518750

**Published:** 2021-10-29

**Authors:** Shuhan Zhou, Xiaohui Xu, Jiangqin Zeng, Zhiyi Liu, Miao Wang, Wenliang Lv

**Affiliations:** Clinical College, Hubei University of Chinese Medicine, Wuhan 430000, China

## Abstract

Lianpu drink (LPD) is a traditional Chinese medicine (TCM) formula for the treatment of chronic gastritis (CG), and its clinical effects have been effectively verified. However, due to the complexity of the chemical composition of TCM formulas, its mechanism of action has not yet been clearly explained. Many studies have shown that the principal drugs in the TCM formula play a major therapeutic role. Therefore, in this study, the principal drugs *Coptidis Rhizoma* (CR) and *Magnolia officinalis Rehd. et Wils*. (MOR) in LPD were used as the main research objects to predict the mechanism of LPD on CG. We contrasted a “compounds-targets-diseases” network and screened the putative targets of CR and MOR in LPD related to CG, respectively. Furthermore, common targets of CR and MOR related to CG were selected as candidate targets. In this study, the specific target proteins of CR, MOR, and CG were combined by protein-protein interaction (PPI) to construct a pharmacological network of “components-targets-diseases.” In addition, we investigated the effects of CR and MOR on the TNF signaling pathway, which mediated the remission of CG. This study preliminarily revealed that CR and MOR play a key role in the treatment of CG. Animal experiments also showed that CR and MOR could significantly improve CG by inhibiting MKK6/*p*38 and RIP/*p*38 pathway.

## 1. Introduction

Chronic gastritis (CG) is the frequent and repeated invasion of gastric mucosal epithelium by various pathogenic factors, resulting in persistent chronic inflammatory changes [1]. Most patients with CG do not have any obvious symptoms. Symptoms are mainly indigestion, which is nonspecific. Some patients with CG may have symptoms of dyspepsia such as epigastric pain and fullness. CG is very common in a clinic, and its incidence rate is the highest among all kinds of stomach diseases, accounting for 40% to 60% of the outpatient clinic. The incidence rate of the disease is generally increased with age, especially in middle age [2]. Although gastritis plays an important role in the pathogenesis of common peptic ulcer and gastric cancer, the importance of CG as a serious disease has been underestimated to a large extent in clinical practice [3–6]. If it is not treated in a timely and effective manner, CG can easily develop into gastric cancer. Gastric cancer is the second most common cancer worldwide after lung cancer. It is estimated that millions of people worldwide may die from cancer and ulcers each year as a consequence of CG [7].

Western medicine mainly through the inhibition of gastric acid secretion, protection of gastric mucosa, and eradication of Helicobacter pylori promote gastrointestinal motility and other measures to treat CG [8]. However, the application of western medicine will lead to bacterial resistance, and drugs cannot effectively control the disease. Therefore, we need to find a safe and effective drug to replace the treatment of western medicine. In recent years, as an effective means to treat CG, Chinese herbal medicine compound has received more and more attention due to its good effects, such as Chaihu-Shugan-San, Dendrobium Yangwei decoction, HuoxueZhitong decoction, BanxiaXiexin decoction, Sanren decoction, and Lianpu drink (LPD) [9–14]. As a traditional Chinese medicine (TCM) compound for the treatment of CG, LPD has a history of several years from the Qin Dynasty to the present. The composition of LPD was *Coptidis Rhizoma* (CR), *Magnolia officinalis Rehd. et Wils*. (MOR), cape jasmine (CJ), *Pinellia ternata* (PT), *Acorus tatarinowii* (AT), fermented soybeans (FSB), and reed rhizome (RR), which is a classic prescription for CG. The clinical effect of LPD in patients with CG has been confirmed, but the mechanism of action, precise targets, and the relationship between CG and disease have not yet been answered. Therefore, further research on LPD is still a big challenge for us.

LPD is an effective TCM formula for the treatment of CG. However, due to the complexity of the chemical composition of TCM, its mechanism of action on diseases has not been clearly explained. Many studies have shown that the principal drugs of TCM play a major role in disease treatment. Therefore, the purpose of our study is to establish a comprehensive and systematic method to predict the mechanism of the principal drugs (CR and MOR) of LPD in the treatment of CG. In this study, the specific target proteins of CR, MOR, and CG were combined by PPI to construct a pharmacological network of “components-targets-diseases.” Meanwhile, several signaling pathways have been proposed to be involved in CG, for example, TLR4/NF-*κ*B/COX-2 signaling [15], TNF signaling pathway, apoptosis, VEGF signaling pathway [16], IL-11/STAT3 signaling pathway [17], and so on. In our study, we investigated the effects of CR and MOR on the TNF signaling pathway, which mediates the remission of CG.

## 2. Materials and Methods

### 2.1. Database Construction

The compounds of “CR” and “MOR” in LPD were obtained from TCM Database@Taiwan (TDT; http://tcm.cmu.edu.tw/zh-tw/review-result.php). Meanwhile, the chemical structures of these compounds were saved in MOL2 format [18]. Subsequently, the predicted targets of the compounds were obtained by uploading the MOL2 format to the online target prediction software of PharmMapper with a criterion of “fit score” >4 (http://www.lilab-ecust.cn/pharmmapper/submitfile.html) [19]. Next, with the “chronic gastritis” as the keywords, the gene and protein targets of disease were collected from the Online Mendelian Inheritance in Man (OMIM) database (https://www.omim.org/) [20]. Finally, the possible interactions of the aforementioned targets were identified by the Database of Interacting Proteins (DIP), and all protein IDs were converted to UniProt IDs [21].

### 2.2. Network Construction and Analysis

To provide a scientific and reasonable interpretation of the complex relationships between the constituents and targets associated with CG, network analysis was performed. The chemical constituents, “CR” and “MOR” putative targets, and CG targets were all connected to construct a “constituent-target-disease” network with PPI information [19]. Cytoscape 7.0 was applied to visualize and analyze the network and calculate the topological features of each node in the network. Only the hub nodes (two folds above the median “degree” value of all nodes) with higher values of “betweenness centrality” and “closeness centrality” (above the median value of all nodes) were identified as the candidate “CR” and “MOR” targets for CG [21].

### 2.3. Targets and Pathways Analyses

To unveil the mechanism of “CR” and “MOR” for treatment of CG, the candidate “CR” and “MOR” targets for CG were uploaded to DAVID Functional Annotation Bioinformatics Microarray Analysis website (https://david.ncifcrf.gov/tools.jsp) for the signal pathway analysis. By focusing on the maximum possible signal pathway, the key target protein in this signaling pathway is identified and verified by performing in vivo experiments.

### 2.4. Experimental Validation

#### 2.4.1. Preparation of Drugs

Classical LPD was composed by weighing 12 g of MOR, 6 g of CR, 6 g of AT, 6 g of PT, 18 g of FSB, 18 g of CJ, and 120 g of RR according to the original formula ratio. To investigate the influence of CR and MOR on LPD, five treatment groups were investigated. The LPD group was composed according to the original formula ratio, that is, weighing 12 g of MOR, 6 g of CR, 6 g of AT, 6 g of PT, 18 g of FSB, 18 g of CJ, and 120 g of RR. The second group was called CR(+) group, the weight of CR was increased to 9 g, and the weights of other ingredients were left unchanged compared with LPD group. The third group was called CR(−) group, the weight of CR was reduced to 3 g, and the weights of other components were kept unchanged compared with LPD group. Similarly, In MOR(+) group, the weight of MOR was increased to 18 g, and the weights of other ingredients were kept unchanged compared with LPD group. In MOR(−) group, the weight of MOR was reduced to 6 g, and the weights of other components were left unchanged compared with LPD group. The above five groups were added 200 mL of water and soaked for 30 min. Then added 200 mL of water, decocted for 15 min, filtered, and concentrated to 186 mL. Obtained classical LPD, CR (+), CR (−), MOR (+), and MOR (−) with a concentration of 1 g/ml and put it in the refrigerator at 4°C for standby.

#### 2.4.2. Animals Modeling and Grouping

Seventy Sprague Dawley (SD) male rats (100–130 g; 3-4 weeks) were purchased from Beijing Weitong Lihua Experimental Animal Technology Co. Ltd., China (SCXK (Jing) 2018–0010) and kept in a standard environment in the lab animal room in clinical college, Hubei University of Chinese Medicine. The CG model in rat was established according to the methods described by previous studies [22, 23]. In brief, the CG rat model was induced by a comprehensive method based on N-methyl-N′-nitro-N-ni-trosoguanidine (MNNG, concentration 150 μg/mL) free drinking, during the experiment, and drinking water needs to be changed every day. Meanwhile, the method of even-numbered days of full food and odd-numbered days of fasting were used to cause the rats hunger and satiety disorder. In even-numbered days, 0.05% Ranitidine's granular SPF grade rat feed was eaten freely. In odd-numbered days, the rats were administered with 0.5 mL/100g of 2% sodium salicylate solution. The normal group was given normal feed and drinking water. The model establishment lasted for 10 weeks.

Rats were randomly divided into seven groups (*n* = 10), namely normal group, model group (CG rats), classical LPD group (hereinafter referred to as LPD group), CR (+) group, CR (−) group, MOR (+) group, and MOR (−) group. After modeling, rats in the normal group were administered with 0.9% normal saline, whereas other groups were administered with 1.5 ml/100 g of different formulas every day, which lasted for 10 weeks.

#### 2.4.3. Hematoxylin-Eosin Staining

Thirty min after the last administration, the stomach of rats in different groups were anesthetized, dissected, and stained with hematoxylin and eosin (H&E) to observe the pathological changes in the stomach. The sections were stained with H&E following a standard protocol of our laboratory. Hematoxylin was applied for 4 minutes followed by a 20 second differentiation in ammonia, after which eosin was applied for 20 seconds.

#### 2.4.4. ELISA Assay

Five milliliter of arterial blood was extracted from the femoral artery of rats, and the serum was separated and stored at −20°C. Meanwhile, seven groups of different rat gastric tissues were stripped, frozen, and preserved. Pepsinogen I (PG I), pepsinogen II (PG II), and gastrin-17 (G-17) ELISA kits were purchased from Shanghai Yuanye Bio-engineering Co. Ltd. (Shanghai, China). Moreover, p-MKK6 (ab280984), RIP3 (ab195117), *p*-*p*38 (ab207483), *p*38 (ab221012), TNF-*α* (ab183218), IFNG (ab239425), IL1B (ab244612), IL6 (ab234570), and IL10 (ab214566) ELISA kits were purchased from Abcam (China). MKK6 (CB13348401) was purchased from Beijing Dongge Boye Biotechnology Co. Ltd. The levels of these factors were measured according to the operating instructions of the kits.

#### 2.4.5. Protein Extraction and Western Blot Analysis

Total proteins were extracted from seven groups of different rat gastric tissues with an animal tissue total protein extraction kit (RIPA buffer, Solarbio, Beijing, China). The protein concentration was determined spectrophotometrically using the Bradford method with serial dilution of bovine serum albumin as the standard. For gel electrophoresis, 20 *μ*g of proteins were used. The samples were separated by SDS-PAGE (10%) at 200 V and 300 mA for 50 min. After transferring the proteins onto polyvinylidene fluoride membranes, the blotting was performed at 200 V and 300 mA for 45 min. After blocking with 5% (w/v) dry milk in TBS for 1 h at room temperature, membranes were incubated with the primary antibodies (RIP3 1:1,000, *p*-*P*38 1:1,000, *P*38 1:1,000, p-MKK6 1:1,000, MKK6 1:1,000, and *β*-actin 1:3,000) at 4°C overnight. Then the membranes were incubated with HRP-conjugated anti-rabbit, anti-goat antibody, or anti-mouse antibody for 2 h at room temperature. In the study, all antibodies were purchased from Abcam, China. Finally, the blots were developed with an enhanced chemiluminescence kit (ECL, CWbio), and the bands were quantified densitometrically using a Bio-Rad imaging system (Hercules, CA). The relative band intensity of each sample was normalized to the *β*-actin signal in the same lane.

### 2.5. Statistical Analysis

Data were represented as mean ± SEM of independent experiments. Statistical analysis was performed using ANOVA and student's *T*-test (two-tailed). *p* < 0.05 were considered significant.

## 3. Results

### 3.1. Targets Screening of “CR” and “MOR” and CG

Based on the database construction (the specific methods can be seen in Section 2.1), 23 compounds and 364 putative targets of “CR,” and 63 compounds and 1,318 putative targets of “MOR” were obtained with a “fit score” >4 by PharmMapper. Meanwhile, 30 protein targets associated with CG therapy were screened out from OMIM. The obtained compounds and targets were all used to construct the “drug-target-disease” network.

### 3.2. Network Construction and Analysis

**Figure 1:**
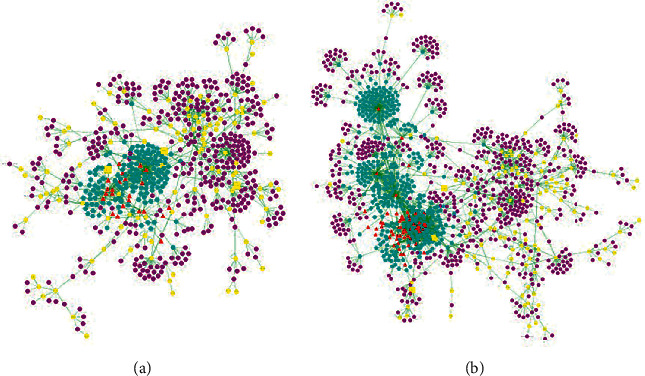
The “drug-target-disease” network for the treatment of CG with CR (a) and MOR (b).

The construction of the “drug-target-disease” network and the analysis of the noteworthy features of the network would provide some important information for us to understand the drug-target interaction mechanism of certain drugs on the specific disease. In this study, we focused on the effects of “CR” and “MOR” on CG. As shown in Figure 1, the network for the compounds and their potential targets was constructed by the nodes with different shapes and colors. The red triangles represent active chemical constituents of “CR” and “MOR”; the blue dots represent the indirect targets for drugs; the yellow dots represent the targets of the specific disease of CG; and the yellow squares represent the common targets of herbs and CG [21].

Based on the network analysis, three topological parameters of “degree,” “betweenness centrality,” and “closeness centrality” were chosen to screen the potential CG targets that “CR” and “MOR” might affect. After calculating the values of the three parameters for each significant protein in the PPI network, the median values of “degree,” “betweenness centrality,” and “closeness centrality” of “CR” were 1, 0, and 0.1668, respectively, and the median values of “degree,” “betweenness centrality,” and “closeness centrality” of “MOR” were 1, 0, and 0.1831, respectively. The protein targets of which the “degree” was more than two folds of the median value and “betweenness centrality” and “closeness centrality” were higher than the median value were chosen as the major targets of “CR” and “MOR” treating CG [19]. As shown in Tables 1 and 2, we finally determined 35 protein targets of “CR” and 26 protein targets of “MOR” for CG therapy. Moreover, there were 14 common target proteins of CR and MOR, including Q16539, P35968, Q99558, P19838, O15350, P09874, Q13546, Q04206, P05412, P25963, P31749, O15111, O14920, and Q9Y6K9.

### 3.3. Targets and Pathways Analyses

**Figure 2:**
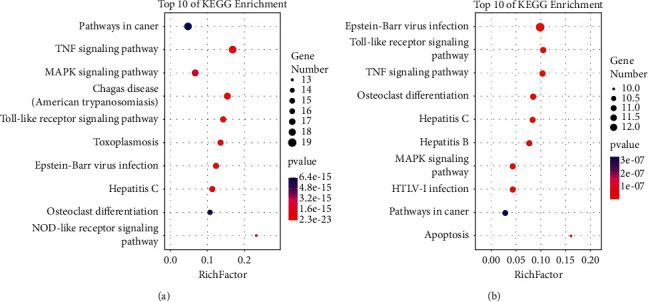
KEGG pathways analysis of “CR” (a) and “MOR” (b) in treating CG.

DAVID Functional Annotation Bioinformatics Microarray Analysis website is a database for annotation, visualization, and integrated discovery, which provides a comprehensive set of functional annotation tools for investigators, such as visualize genes on BioCarta and KEGG pathway maps, to understand biological meaning behind a large list of genes [24]. With the candidate “CR” and “MOR” targets for CG uploaded, the top 10 signaling pathways of “CR” and “MOR” were selected. As shown in Figure 2, the TNF signaling pathway is ranked in the top three in both “CR” and “MOR.” Moreover, we investigated the relevant literature on the TNF signaling pathway [25, 26] and found that there was a close connection between TNF signaling pathway and chronic gastritis. Therefore, in our study, TNF signaling pathway and its contained protein targets were chosen for further research. Based on the analysis of KEGG, there were 17 targets of “CR” and 11 targets of “MOR” involved in the TNF signaling pathway respectively (as shown in Table 3). Furthermore, network pharmacology analysis results demonstrated that there were three common targets of CR and CG, including adenine phosphoribosyltransferase (No. P07741, gene name APRT), mitogen-activated protein kinase 14 (No. Q16539, gene name MAPK14), and DNA repair protein complementing XP-G cells homolog (No. P35689, gene name Ercc5). Meanwhile, mucin-1 (No. P15941, gene name MUC1), mitogen-activated protein kinase 14 (No. Q16539, gene name MAPK14), and vascular endothelial growth factor receptor 2 (No. P35968, gene name KDR) were the common targets of MOR and CG. The results showed that MAPK14 (*p*38) was the common target between CR, MOR, and CG.

### 3.4. Effects of the Principal Drugs of LPD on CG

**Figure 3:**
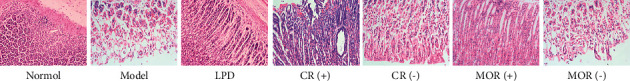
HE staining of gastric tissues in different groups: (a) normal, (b) model, (c) LPD, (d) CR(+), (e) CR(−), (f) MOR(+), and (g) MOR(−).

**Figure 4:**
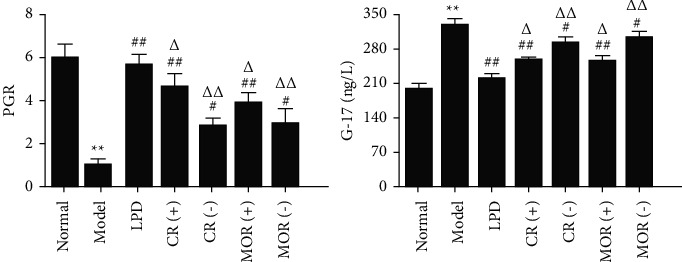
Effects of the addition or subtraction of CR or MOR in classical LPD on CG rats. The levels of PGR and G-17 in serums from different groups were detected by ELISA. Model groups (CG rats) compared with the normal groups, ^*∗*^*p* < 0.05 and ^*∗∗*^*p* < 0.01. Different formulas treatment groups compared with the model groups, ^#^*p* < 0.05 and ^##^*p* < 0.01. The addition or subtraction of CR or MOR treatment groups compared with LPD groups, ^Δ^*p* < 0.05 and ^ΔΔ^*p* < 0.01.

HE staining (Figure 3) showed that the glandular structure of gastric mucosa in the normal group was clear, without obvious degeneration, necrosis, and inflammatory cell infiltration, and the mucosal muscle structure was clear. In the model group, the lamina propria of gastric mucosa became thinner; the cells were arranged in disorder; the gland structure was disordered and atrophied; the gland cavity was enlarged; the epithelial vacuoles were expanded; a large number of inflammatory cells were infiltrated; and the glands were destroyed obviously. Moreover, to further verify the effects of the addition or subtraction of CR or MOR of LPD on CG, the levels of PG I, PG II, and G-17 in different groups were detected in this study. The results indicated that compared to the normal group, the rate of PG I to PG II (PGR) was significantly decreased, while the G-17 level was obviously increased in the model group (Figure 4). The results showed the CG model was successfully established.

Furthermore, compared with the model group, the thickness of gastric mucosal lamina propria in the LPD group was significantly restored, the degree of gland atrophy was significantly reduced; the mucosal epithelial cells were basically complete; and the inflammatory cell infiltration in the mucosal layer was not obvious. Compared with the model group, the thickness of lamina propria of gastric mucosa gradually recovered in CR (+) and MOR (+) groups; the surface epithelial cells were continuous; and the infiltration of inflammatory cells was significantly reduced. Compared with the LPD group, some inflammatory cells still infiltrated in the mucosal layer of CR (+) and MOR (+) groups. Besides, compared with the model group, the structural disorder and atrophy of gastric mucosal glands in CR (−) and MOR (−) groups were slightly improved; there were still a large number of inflammatory cell infiltration; and the thickness of gastric mucosal lamina propria was thickened in varying degrees. Compared with the LPD group, the thickness of gastric mucosal lamina propria in CR (−) and MOR (−) groups did not recover well, and there was still obvious infiltration of inflammatory cells in the mucosal layer. Moreover, PGR was significantly improved, while G-17 expression was remarkably decreased by different formulas in varying degrees. Meanwhile, the addition or subtraction of CR or MOR of LPD significantly reduced the therapeutic effects of LPD on CG. It can be seen that LPD significantly improved CG. CR (+) and MOR (+) have the second effect; CR (−) and MOR (−) have a little effect but are not very obvious. The results showed that the doses of CR and MOR were not directly proportional to the therapeutic effects of CG. It is worth mentioning that the addition or subtraction of the dose of CR and MOR on traditional LPD significantly weakened the therapeutic effect of LPD on CG. These results demonstrated that CR and MOR were the principal drugs of LPD on CG indeed.

### 3.5. Effects of DR and MOR on TNF Signaling Pathway

**Figure 5:**
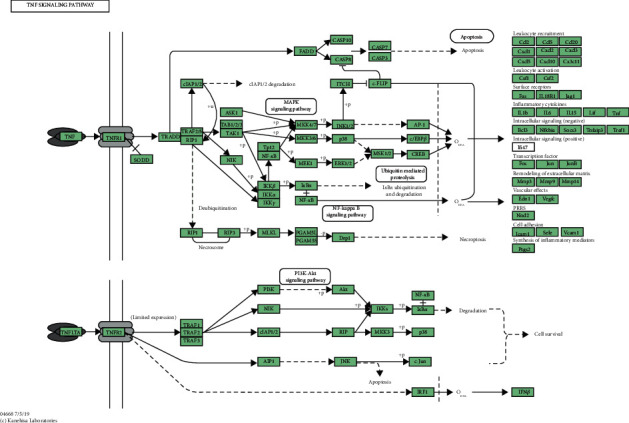
KEGG pathways analysis of TNF signaling pathway (supplementary data).

**Figure 6:**
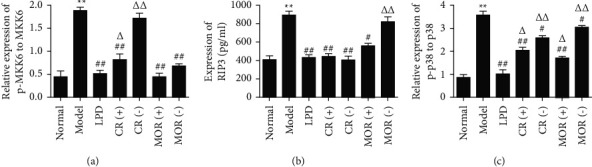
Effects of the addition or subtraction of CR or MOR in classical LPD on TNF signaling pathway in CG rats gastric tissues detected by ELISA. Model groups compared with the normal groups, ^*∗*^*p* < 0.05 and ^*∗∗*^*p* < 0.01. Different formulas treatment groups compared with the model groups, ^#^*p* < 0.05 and ^##^*p* < 0.01. The addition or subtraction of CR or MOR treatment groups compared with LPD groups, ^Δ^*p* < 0.05 and ^ΔΔ^*p* < 0.01.

**Figure 7:**
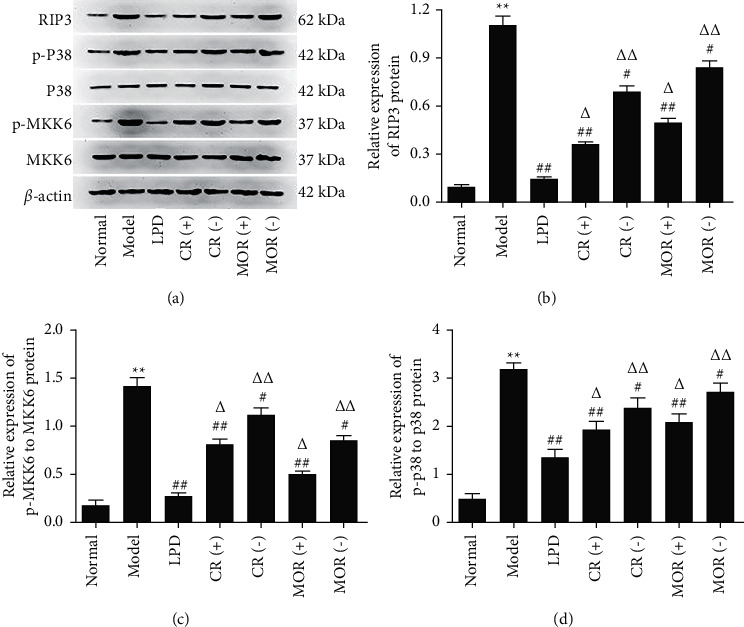
Effects of the addition or subtraction of CR or MOR in classical LPD on TNF signaling pathway in CG rats gastric tissues verified by western blotting. Model groups compared with the normal groups, ^*∗*^*p* < 0.05 and ^*∗∗*^*p* < 0.01. Different formulas treatment groups compared with the model groups, ^#^*p* < 0.05 and ^##^*p* < 0.01. The addition or subtraction of CR or MOR treatment groups compared with LPD groups, ^Δ^*p* < 0.05 and ^ΔΔ^*p* < 0.01.

As shown in supplementary data (Figure 5), according to the KEGG analysis of the TNF signaling pathway, MKK6/*p*38 and RIP/*p*38 pathway got our attention. What is interesting was that MKK6 and RIP were precisely the prediction targets of CR and MOR in the TNF signaling pathway, respectively. In order to investigate whether CR and MOR play a role in LPD alleviate CG by changing the disease targets through acting on different target proteins in TNF signaling pathway, we measured the expressions of p-MKK6, MKK6, RIP, *p*-*p*38, and *p*38 by ELISA and western blotting. As shown in Figures 6 and 7, compared with normal groups, the relative levels of p-MKK6 to MKK6, *p*-*p*38 to *p*38, and expression of RIP3 in model groups were significantly increased. Furthermore, compared with model groups, LPD remarkably decreased the relative levels of p-MKK6 to MKK6, *p*-*p*38 to *p*38, and expression of RIP3. It is worth noting that the addition or subtraction of the dose of CR or MOR alone significantly increased the relative levels of p-MKK6 to MKK6 and *p*-*p*38 to *p*38 compared with the LPD group, respectively. The results demonstrated that 6 g of CR and 12 g of MOR in classical LPD may inhibit the phosphorylation of MKK6, and the activation of RIP3 thereby prevented *p*38 phosphorylation to relieve CG, while the addition and subtraction of CR or MOR both affected the activating of TNF signaling pathway.

**Figure 8:**
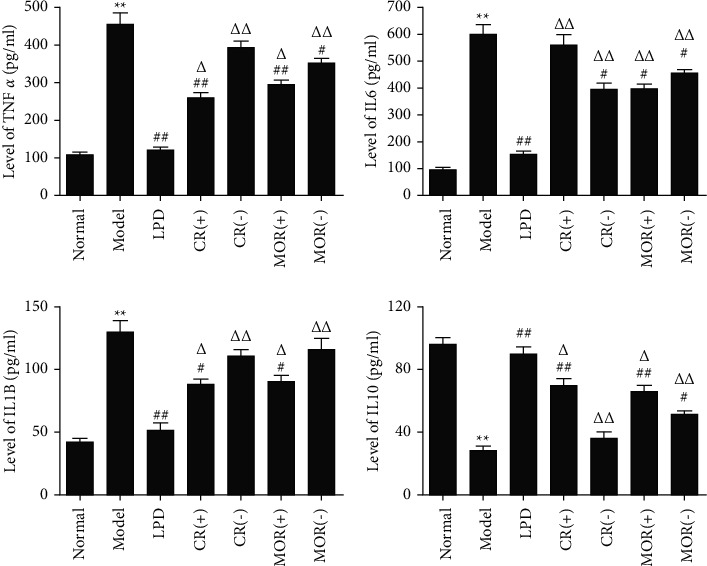
Effects of the addition or subtraction of CR or MOR in classical LPD on downstream targets of TNF signaling pathway. The levels of TNF-*α*, IL6, IL1B, and IL10 in serums from different groups were detected by ELISA. Model groups compared with the normal groups, ^*∗*^*p* < 0.05 and ^*∗∗*^*p* < 0.01. Different formulas treatment groups compared with the model groups, ^#^*p* < 0.05 and ^##^*p* < 0.01. The addition or subtraction of CR or MOR treatment groups compared with LPD groups, ^Δ^*p* < 0.05 and ^ΔΔ^*p* < 0.01.

To further evaluate the expressions of downstream target proteins related to the TNF signaling pathway, expressions of TNF-*α*, IL6, IL1B, and IL10 were detected. Results showed that compared with normal groups, the expressions of TNF-*α*, IL1B, and IL6 were significantly increased, while IL10 was obviously decreased in CG rats (Figure 8). Moreover, compared with model groups, LPD significantly reduced TNF-*α*, IL1B, and IL6 expressions while remarkably improved the levels of IL10. In addition, the addition or subtraction of CR or MOR of LPD inhibited the effects of LPD on the expressions of downstream target proteins related to the TNF signaling pathway in varying degrees.

## 4. Discussion

CG, as an inflammatory condition of the gastric mucosa, has infected more than half of people in the world [7, 26]. Its clinical performance is mental sluggishness, anorexia, acid reflux, belching, and so on; if it is not treated in a timely and effective manner, CG can easily develop into gastric cancer [27, 28]. At present, CG is usually treated with chemicals such as ranitidine and omeprazole; however, these chemicals have big side effects; after taking for a long time, the liver and kidney function of the human body will be damaged to some degree [29, 30]. Therefore, it is necessary to find a drug that is safe and effective and can reveal its mechanism of action on CG. As crystallization of the wisdom of the Chinese Nation, Chinese Traditional Medicine Compound has an advantage in the treatment of disease [31].

LPD, as a classic prescription for the treatment of CG, consists of seven herb medicine, namely CR, MOR, CJ, PT, AT, FSB, and RR. In the TCM compound, the sovereign drug plays a major role in the treatment of the main disease [31]. So, in our study, “CR” and “MOR” as sovereign drugs of LPD had been taken for research. To uncover the therapeutic effect of “CR” and “MOR” on CG, an integrated model of system pharmacology was structured. The “components-targets-diseases” network of “CR” and “MOR” related to CG indicated that 35 protein targets of CR and 26 protein targets of MOR were interacted with 30 targets related to CG in the network. Meanwhile, 14 targets in the aforementioned predicted targets are the same. It indicated that “CR” and “MOR” exert their therapeutic effects through the synergistic effects of multiple targets; the prediction results were consistent with the guess that the herbal medicines exert their therapeutic effects through the synergistic effects of multiple compounds and targets [32].

To further determine the mechanism of principal drugs of LPD on CG, KEGG pathways analysis and animal experiments have been applied. As shown in Figure 2 and Table 3, 17 targets of “CR” and 11 targets of “MOR” are involved in the TNF signaling pathway. In the above targets, MAPK14 (*p*38) was the common target between CR, MOR, and CG. It indicated that “CR” and “MOR” may exert their therapeutic effects by acting on MAPK14 (*p*38) target. So, in our study, we further investigated how the CR and MOR acted on MAPK14 (*p*38) target in TNF signaling pathway. As shown in Figures 6 and 7, we finally found that CR and MOR may inhibit the phosphorylation of MKK6, and the activation of RIP3 thereby prevented *p*38 phosphorylation. To further evaluate the expressions of downstream target proteins related to the TNF signaling pathway, expressions of TNF-*α*, IL6, IL1B, and IL10 were detected. Results showed that the addition or subtraction of CR or MOR of LPD inhibited the effects of LPD on the expressions of downstream target proteins related to the TNF signaling pathway in varying degrees. These results indicated that the metabolism of MAPK14 (*p*38) and its downstream target proteins was involved in the development of CG, of which the stabilization could be regulated by CR and MOR of LPD.

In our study, we found that CR (+)/(−) and MOR (+)/(−) can alleviate CG to varying degrees, and the therapeutic effect of CR (+)/MOR (+) were better than CR (−)/MOR (−), respectively. This was proved by the consistent results of HE, PGR, G-17, the relative levels of p-MKK6 to MKK6 and *p*-*p*38 to *p*38 (both ELISA and WB), RIP3 (WB), TNF-*α*, IL-1B, and IL-10. Of course, the consistency between RIP3 in gastric tissue and IL6 in serum measured by ELISA and other indexes were indeed a little weak. However, this does not affect our conclusion that increasing or reducing the doses of CR and MOR were not as good as the classical LPD formula. Interestingly, the classical doses of CR and MOR in LPD were between CR (+)/MOR (+) and CR (−)/MOR (−), but the efficacy of LPD was the best. In order to explain this trend, we investigated the relevant literature [33, 34] and found that many TCM have mutual regulatory effects due to different doses. In the case of *Astragalus membranaceus*, a small amount (less than 15 g) can increase blood pressure. On the contrary, a large amount (more than 30 g) inhibited the therapeutic effect. Our results indicated that 6 g of CR and 12 g of MOR play key roles in classical LPD. It was not just a large dose but also a good effect. Their contribution as principal drugs may be that CR and MOR interact, cooperate, or inhibit with other drugs at a specific dose in classical LPD, so as to achieve a better therapeutic effect on CG. This fully reflects that the efficacy of TCM is the result of the complex coaction of multiple components and multiple targets. We speculate that the addition or subtraction of CR and MOR may have an unknown impact on the interaction between other components and between components and targets. Finding the answers to these questions is also the focus of our next work.

## 5. Conclusion

CR and MOR can alleviate CG through the molecular mechanism predicted by network pharmacology. In addition, network pharmacology can provide an in-depth understanding of the pharmacological mechanism of Chinese herbal formulas. In this study, we firstly predicted the potential targets of CR and MOR related to the treatment of CG disease by constructing a “compounds-targets-diseases” interaction network. The results showed that “CR” and “MOR” may exert their therapeutic effects by acting on MAPK14 (*p*38) target. In addition, with the KEGG pathways analysis and animal experiments, we have found that CR and MOR may inhibit the phosphorylation of MKK6 and the activation of RIP3, thereby preventing *p*38 phosphorylation. Although we have clarified the mechanism of principal drugs of LPD on CG, there were some limitations in the present study. This study only validated TNF signaling pathway; however, other biological signaling pathways have not been conducted in in-depth studies. Therefore, additional studies on the possible pathways of CR and MOR in the treatment of CG will be further carried out.

## Figures and Tables

**Table 1:** The information of target proteins of “CR” predicted by network pharmacology analyses.

Uniprot no.	Protein name	Gene name	Closeness centrality	Degree	Betweenness centrality
Q9Y6K9	NF-kappa-B essential modulator	IKBKG	0.2195	23	0.0539
Q9Y4K3	TNF receptor-associated factor 6	TRAF6	0.1998	22	0.0686
P35222	Catenin beta-1	CTNNB1	0.1781	22	0.0565
P19838	Nuclear factor NF-kappa-B *p*105 subunit	NFKB1	0.2001	21	0.0360
P00533	Epidermal growth factor receptor	EGFR	0.1994	19	0.0594
Q04206	Transcription factor *p*65	RELA	0.2037	19	0.0255
O15111	Inhibitor of nuclear factor kappa-B kinase subunit alpha	CHUK	0.2225	18	0.0790
P25963	NF-kappa-B inhibitor alpha	NFKBIA	0.2163	18	0.0391
O14920	Inhibitor of nuclear factor kappa-B kinase subunit beta	IKBKB	0.2150	15	0.0182
P05412	Transcription factor AP-1	JUN	0.2060	13	0.0580
P31749	RAC-alpha serine/threonine-protein kinase	AKT1	0.2009	13	0.0344
Q99558	Mitogen-activated protein kinase kinasekinase 14	MAP3K14	0.2037	13	0.0207
P25445	Tumor necrosis factor receptor superfamily member 6	FAS	0.1964	11	0.0725
P09874	Poly [ADP-ribose] polymerase 1	PARP1	0.2071	11	0.0374
P35968	Vascular endothelial growth factor receptor 2	KDR	0.1910	11	0.0350
P19438	Tumor necrosis factor receptor superfamily member 1A	TNFRSF1A	0.1718	11	0.0237
Q99759	Mitogen-activated protein kinase kinasekinase 3	MAP3K3	0.1793	10	0.0235
O15350	Tumor protein *p*73	TP73	0.1954	9	0.0152
P04150	Glucocorticoid receptor	NR3C1	0.1796	8	0.0323
Q13546	Receptor-interacting serine/threonine-protein kinase 1	RIPK1	0.2052	7	0.0935
Q14790	Caspase-8	CASP8	0.1748	6	0.0102
P41279	Mitogen-activated protein kinase kinasekinase 8	MAP3K8	0.1714	6	0.0000
Q9HC29	Nucleotide-binding oligomerization domain-containing protein 2	NOD2	0.1747	5	0.0118
Q13191	E3 ubiquitin-protein ligase CBLB	CBLB	0.1984	5	0.0088
P29466	Caspase-1	CASP1	0.1750	4	0.0263
Q16236	Nuclear factor erythroid 2-related factor 2	NFE2L2	0.1971	4	0.0088
Q30201	Hereditary hemochromatosis protein	HFE	0.1976	3	0.0205
P35869	Aryl hydrocarbon receptor	AHR	0.1822	3	0.0191
P55957	BH3-interacting domain death agonist	BID	0.1862	3	0.0073
P35228	Nitric oxide synthase	NOS2	0.1970	3	0.0059
P49281	Natural resistance-associated macrophage protein 2	SLC11A2	0.1970	3	0.0059
P40763	Signal transducer and activator of transcription 3	STAT3	0.1725	3	0.0059
P45983	Mitogen-activated protein kinase 8	MAPK8	0.1813	3	0.0047
P45984	Mitogen-activated protein kinase 9	MAPK9	0.1721	3	0.0043
Q16539	Mitogen-activated protein kinase 14	MAPK14	0.1921	3	0.0039

**Table 2:** The information of target proteins of “MOR” predicted by network pharmacology analyses.

Uniprot no.	Protein name	Gene name	Closeness centrality	Degree	Betweenness centrality
P41182	B-cell lymphoma 6 protein	BCL6	0.2056	8	0.0369
P17535	Transcription factor jun-D	JUND	0.1857	4	0.0023
P15941	Mucin-1	MUC1	0.2361	3	0.0339
Q14164	Inhibitor of nuclear factor kappa-B kinase subunit epsilon	IKBKE	0.1967	5	0.0052
Q16539	Mitogen-activated protein kinase 14	MAPK14	0.2029	5	0.0022
Q03164	Histone-lysine N-methyltransferase 2A	KMT2A	0.1989	5	0.0056
P35968	Vascular endothelial growth factor receptor 2	KDR	0.1856	11	0.0189
Q99558	Mitogen-activated protein kinase kinasekinase 14	MAP3K14	0.1887	13	0.0111
P19838	Nuclear factor NF-kappa-B *p*105 subunit	NFKB1	0.1959	21	0.0189
P04637	Cellular tumor antigen *p*53	TP53	0.2289	60	0.1438
O15350	Tumor protein *p*73	TP73	0.1879	9	0.0037
P09874	Poly [ADP-ribose] polymerase 1	PARP1	0.2023	11	0.0449
P60900	Proteasome subunit alpha type-6	PSMA6	0.1846	3	0.0022
Q13546	Receptor-interacting serine/threonine-protein kinase 1	RIPK1	0.1833	7	0.0475
Q86WV6	Stimulator of interferon genes protein	STING1	0.1851	8	0.0049
Q04206	Transcription factor *p*65	RELA	0.1974	19	0.0133
P24385	G1/S-specific cyclin-D1	CCND1	0.2329	10	0.0877
P05412	Transcription factor AP-1	JUN	0.2135	13	0.0278
P25963	NF-kappa-B inhibitor alpha	NFKBIA	0.2177	18	0.0477
P06396	Gelsolin	GSN	0.2326	5	0.0276
P31749	RAC-alpha serine/threonine-protein kinase	AKT1	0.2153	13	0.0323
Q9C000	NACHT, LRR and PYD domains-containing protein 1	NLRP1	0.1853	4	0.0141
Q16665	Hypoxia-inducible factor 1-alpha	HIF1A	0.2139	12	0.0151
O15111	Inhibitor of nuclear factor kappa-B kinase subunit alpha	CHUK	0.2166	18	0.0318
O14920	Inhibitor of nuclear factor kappa-B kinase subunit beta	IKBKB	0.2158	15	0.0206
Q9Y6K9	NF-kappa-B essential modulator	IKBKG	0.2163	23	0.0446

**Table 3:** Predicted target proteins of CR and MOR involved in the TNF signaling pathway.

Drugs	Uniprot no.	Protein name	Gene name
CR	P31749	RAC-alpha serine/threonine-protein kinase	AKT1
	P25445	Tumor necrosis factor receptor superfamily member 6	FAS
	P05412	Transcription factor AP-1	JUN
	P25963	NF-kappa-B inhibitor alpha	NFKBIA
	Q04206	Transcription factor *p*65	RELA
	P19438	Tumor necrosis factor receptor superfamily member 1A	TNFRSF1A
	Q14790	Caspase-8	CASP8
	O15111	Inhibitor of nuclear factor kappa-B kinase subunit alpha	CHUK
	P52564	Mitogen-activated protein kinase kinase6	MKK6
	Q9Y6K9	NF-kappa-B essential modulator	IKBKG
	Q16539	Mitogen-activated protein kinase 14	MAPK14
	P45983	Mitogen-activated protein kinase 8	MAPK8
	P45984	Mitogen-activated protein kinase 9	MAPK9
	Q99558	Mitogen-activated protein kinase kinasekinase 14	MAP3K14
	P41279	Mitogen-activated protein kinase kinasekinase 8	MAP3K8
	P19838	Nuclear factor NF-kappa-B *p*105 subunit	NFKB1
	Q9HC29	Nucleotide-binding oligomerization domain-containing protein 2	NOD2

MOR	P31749	RAC-alpha serine/threonine-protein kinase	AKT1
	P05412	Transcription factor AP-1	JUN
	P25963	NF-kappa-B inhibitor alpha	NFKBIA
	Q04206	Transcription factor *p*65	RELA
	O15111	Inhibitor of nuclear factor kappa-B kinase subunit alpha	CHUK
	O14920	Inhibitor of nuclear factor kappa-B kinase subunit beta	IKBKB
	Q9Y6K9	NF-kappa-B essential modulator	IKBKG
	Q16539	Mitogen-activated protein kinase 14	MAPK14
	Q99558	Mitogen-activated protein kinase kinasekinase 14	MAP3K14
	P19838	Nuclear factor NF-kappa-B *p*105 subunit	NFKB1
	Q13546	Receptor-interacting serine/threonine-protein kinase 1	RIPK1

## Data Availability

All data used to support the findings of this study are available from the corresponding author upon request.

## Abbreviations

LPD: Lianpu drink
TCM: Traditional Chinese medicine
CG: Chronic gastritis
CR: *Coptidis Rhizoma*
MOR: *Magnolia officinalis Rehd. et Wils*
CJ: Cape jasmine
PT: *Pinelliaternata*
AT: *Acorus tatarinowii*
FSB: Fermented soybeans
RR: Reed rhizome
TDT: TCM database@Taiwan
OMIM: Online mendelian inheritance in man (OMIM) database
DIP: Database of interacting proteins
KEGG: Kyoto encyclopedia of genes and genomes
PG: Pepsinogen
G-17: Gastrin-17
PGR: Rate of PG I to PG II
TNF: The tumor necrosis factor
DD: Recruitment of death domain
FADD: Fas-associated death domain
TRADD: TNFR-associated DD.
